# Tofacitinib, a suppressor of NOD2 expression, is a potential treatment for Blau syndrome

**DOI:** 10.3389/fimmu.2023.1211240

**Published:** 2023-06-21

**Authors:** Yoko Ueki, Riko Takimoto-Ito, Megumu K. Saito, Hideaki Tanizaki, Naotomo Kambe

**Affiliations:** ^1^ Department of Dermatology, Kansai Medical University, Hirakata, Japan; ^2^ Department of Dermatology, Kyoto University Graduate School of Medicine, Kyoto, Japan; ^3^ Department of Clinical Application, Center for iPS Cell Research and Application, Kyoto University, Kyoto, Japan

**Keywords:** autoinflammatory disease, Blau syndrome, *NOD2*, JAK-STAT signaling pathway, tofacitinib, IFNγ

## Abstract

**Introduction:**

Blau syndrome is a rare autosomal dominant autoinflammatory granulomatous disease caused by a mutation in the *NOD2* gene. It is characterized by a clinical trial of granulomatous dermatitis, arthritis, and uveitis. Tofacitinib is a pan Janus kinase (JAK) inhibitor used for treatment of Blau syndrome and idiopathic sarcoidosis. Here, we evaluated its effect on inflammatory pathways associated with Blau syndrome. The effect of tofacitinib on downstream pathways regulated by mutant *NOD2* was analyzed using luciferase assays with overexpression of *NOD2* mutants.

**Methods:**

The effect of tofacitinib on the upstream pathway for the induction of *NOD2* expression and proinflammatory cytokine production was assessed using monocytic cell lines differentiated from Blau syndrome patient-derived induced pluripotent stem cells.

**Results:**

Tofacitinib did not suppress the increased spontaneous transcriptional activity of NF-κB by mutant *NOD2*. In addition, mutant *NOD2* was not involved in the transcription of ISRE and GAS, which are activated by type 1 and type 2 interferons (IFN), respectively. On the other hand, IFNγ induced the expression of *NOD2*, which led to the production of inflammatory cytokines by an autoinflammatory mechanism only in cells with mutant *NOD2*.

**Discussion:**

Tofacitinib suppressed the induction of *NOD2* by IFNγ, thereby inhibiting the production of pro-inflammatory cytokines. Thus, tofacitinib showed anti-inflammatory effects through suppression of *NOD2* expression. The JAK inhibitor tofacitinib is a potential therapeutic agent for Blau syndrome because it suppresses the autoinflammation seen in Blau syndrome by inhibiting the expression of *NOD2*.

## Introduction

Sarcoidosis is a systemic inflammatory disease of unknown etiology that is characterized by granulomas in multiple organs. Less than 0.5% of all patients with sarcoidosis demonstrate distinct clinical manifestations, with onset before the age of 4 years, in the skin, joints, and eyes, without bilateral hilar lymphadenopathy (BHL). It was previously referred to as early-onset sarcoidosis (EOS) (1). Unlike sarcoidosis in older patients, EOS is progressive and causes severe complications such as joint destruction and blindness (2, 3). Heterozygous mutations in the nucleotide-binding oligomerization domain 2 gene (*NOD2*) were identified as the cause of this syndrome based on both familial and sporadic cases (1, 4, 5). This distinct pathology is referred to as Blau syndrome (6).

The NOD2 protein contains 3 domain structures: 2 sets of N-terminal caspase activation and recruitment domains (CARD) as signal transduction domains and C-terminal leucine-rich repeats (LRR) (7, 8). The domain located in the center is called the NACHT domain, named after the structure commonly found in NAIP, CIITA, HET-E, TEP1, and is evolutionarily conserved. This NACHT domain is also called the NOD domain in NLR family molecules. NOD2 works as an intra-cellular pattern recognition receptor where it recognizes muramyl dipeptide (MDP), a bacterial cell-wall component, leading to the activation of the nuclear factor κB (NF-κB) pathway. Activation causes the production and release of inflammatory cytokines and chemokines. Mutations in *NOD2* are associated with two granulomatous disorders, Blau syndrome and Crohn disease. In patients with Crohn disease, a common bowel inflammatory disorder, loss-of-function mutations have been identified mainly in the LRR of NOD2 (9), whereas in patients with Blau syndrome mutations are in the centrally located NOD domain. In overexpression systems using plasmids expressing Blau syndrome-associated NOD2 mutants transfected into HEK293 cells, ligand-independent NF-κB activation was observed (1, 10, 11). Thus, the NOD2 mutations identified in Blau syndrome are likely gain-of-function mutations.

The third autoinflammatory disorder involving mutations in *NOD2*, Yao syndrome, has also been described, which is characterized by mutations in the intron of the *NOD2* gene, IVS8 + ^158^, and may be associated with missense mutations in the NOD domain that differ from those in Blau syndrome (12–15). Yao syndrome is an independent disease from Blau syndrome because of the absence of uveitis, joint deformities, and granulomas on skin biopsy, although it presents with clinical symptoms such as periodic fever, dermatitis, polyarthritis, and serositis (12). Yao syndrome also differs from the clinical course of Blau syndrome in that it is an adult-onset, solitary disease. In this syndrome, a mutation in *NOD2* is not responsible for the disease, but is presumed to be involved in the amplification and modification of inflammation (13).

We have been working on the analysis of Blau syndrome and have established induced pluripotent stem (iPS) cells from Blau syndrome patients. Functional analyses using these iPS cells revealed that interferon (IFN)γ is an important mediator for the initiation of the inflammatory development in Blau syndrome (16). This experimental finding is supported by the clinical observation that bacillus Calmette–Guesrin (BCG) vaccination is sometimes associated with disease onset (10, 17), since IFNγ is a major cytokine associated with BCG-mediated immune responses. However, the detailed molecular mechanisms of NOD2 mutations leading to granuloma formation remain unclear. Hence, no specific treatment for Blau syndrome has been established.

In 2018, Damsky et al. (18) reported a patient with cutaneous sarcoidosis who was successfully treated with tofacitinib, a pan Janus kinase (JAK) inhibitor, which acts mostly as a JAK1/3 inhibitor and has low/slight effect on JAK2. They suggested that JAK/signal transducer and activator of transcription (STAT) signaling plays a role in the pathogenesis of sarcoidosis. IFNγ activates the JAK/STAT signaling pathway, resulting in the up-regulation of STAT1 transcriptional targets. Several studies have shown that activation signatures of the JAK-STAT pathway, especially STAT1-dependent transcripts, are characteristic of the transcriptome in both peripheral blood mononuclear cells and other tissues in patients with sarcoidosis. They also reported that JAK inhibitors are effective in other granulomatous skin diseases (19). Another group in China recently reported the successful treatment of three Blau syndrome patients with tofacitinib (20). Therefore, in this study, we evaluated the effect of tofacitinib on inflammatory pathways associated with Blau syndrome as a potential therapeutic approach for the treatment of Blau syndrome.

## Materials and methods

### Luciferase assay

The activities of *NOD2* mutants were evaluated by dual luciferase reporter assays, as described previously (1, 10, 11). Briefly, mutants were generated using the QuikChange site-directed mutagenesis kit (Stratagene, La Jolla, CA) and subcloned into the p3xFLAG-CMV vector (Sigma-Aldrich, St. Louis, MO). Two consensus elements in the IFN-stimulated gene promoters responsible for IFN induction have been detected: the ISRE (IFN-stimulated response element) for IFN-α/β activation and the GAS (IFN-γ-activated site) for IFN-γ activation (21). The plasmid of pLuc-MCS (Stratagene) containing 4 copies of the GAS element (5’-AGT GAT TTC TCG GAA AGA GAG-3’) or ISRE element (5′-CGA AGT ACT TTC AGT TTC ATA TTA GG-3′) (22) were kindly provided from Drs. Y. Takahashi and Y. Takakura (Kyoto University).

HEK293FT cells were transfected with a pGreenFire NF-kB reporter vector (System Biosciences, Mountain View, CA) or ISRE or GAS reporters, along with wild type (WT) or mutant *NOD2* and as an internal control *Renilla* gene under phRL-Tk vector (Promega, Madison, WI). Transfected HEK293FT cells were cultured with or without 10 μg/mL of MDP (InvivoGen, San Diego, CA) and/or 1 μM tofacitinib (Sigma-Aldrich) for 24 h. As a positive control for the ISRE or GAS luciferase assay, cells were cultured with 1000 U/mL IFNα (PBL Assay Science, Piscataway, NJ) or 50 ng/mL IFNγ (PeproTech, Cranbury, NJ), respectively. The results were expressed as the ratio of firefly to *renilla* luciferase activity, and we set the activity of untreated cells transfected with mock vector as the standard.

### Patient-derived iPS cells

Patient-derived iPS cells were established from peripheral blood cells from a Blau syndrome patient who had a p.R334W mutation in *NOD2*, as described previously (16). We also corrected the mutation in iPS cell clones by using CRISPR-Cas9–mediated targeting. Differentiation into the monocytic cell lineage was described previously (16). Immortalized proliferating monocytic cell lines (iPS-ML) were then established by introducing 3 transgenes (*cMYC*, *BMI1*, and *MDM2*) into the CSII-EF-RfA vector. They were kindly provided by Drs S. Senju (Kumamoto University) and H. Miyoshi (RIKEN Bio Resource Center), as also described previously (16). Then, iPS-ML cells were maintained in StemPro-34 serum-free medium (Thermo Fisher Scientific, Waltham, MA) containing 2 mmol/l L-glutamine with M-CSF (50 ng/mL, R&D Systems, Minneapolis, MN).

### Quantitative polymerase chain reaction

iPS-ML cells (1x10^6^/mL) were cultured with 50 ng/mL IFNγ for 6 h. In some cases, 1 μM tofacitinib was added. RNA was prepared with the RNeasy Mini Kit (QIAGEN, Hilden, Germany) and subjected to reverse transcription with a PrimeScript RT reagent kit (Takara Bio, Shiga, Japan). qPCR was performed on the Light Cycler 480 System (Roche, Basel Switzerland). qPCR data were processed using the ΔΔcycle threshold method, and the quantities relative to the untreated sample are shown. The primers used in this study are listed in **Table 1**.

**Table 1:** List of primers.

*NOD2*	GGGGTTTCGTCAGCCAGTAT	ATGTGGCAGCTTCCAAAGGC
*TNFA*	TGCTTGTTCCTCAGCCTCTT	GGTTTGCTACAACATGGGCT
*IL8*	AAGGGCCAAGAGAATATCCG	AAACCAAGGCACAGTGGAAC
*GAPDH*	CCTGGTATGACAACGAATTTGGC	TCTCTTCCTCTTGTGCTCTTGC

### Cytokine assay

Concentrations of cytokines in the culture supernatants were measured by using a human IL-8/CXCL8 DuoSet ELISA (R&D system) with DuoSet Ancillary Reagent Kit 2 (R&D system) in accordance with the manufacturer’s instructions. Quantification was done with a FlexStation3 (Molecular Devices, San Jose, CA).

### Immunoblotting

The iPS-ML cells (1x10^6^/mL) were cultured with 50 ng/ml of IFNγ for the time exhibited. In some cells, 1 µM tofacitinib were added. Cells were lysed with dithiothreitol and incubated at 95°C for 5 min. Proteins were then separated by using SDS-PAGE and transferred onto a polyvinylidene difluoride membrane. After blocking with Blocking buffer (nacalai tesque, Kyoto, Japan), immunoblotting was performed with monoclonal antibodies to phospho-STAT1 (Cell Signaling Technology, Danvers, MA) and β-actin (MBL, Tokyo, Japan) and exposed with ECL Western Blotting Reagent (Cytiva, Marlborough, MA).

### Statistics

After confirming the superiority and normality distribution by two-way ANOVA, the Tukey method was used for multiple comparisons. All of the statistical analyses were performed with Prism software (GraphPad Software, La Jolla, CA). The values are presented as means ± the standard deviation, as indicated in the figure legends.

## Results

### Tofacitinib does not inhibit spontaneous NF-κB hyperactivation by mutant NOD2

**Figure 1:**
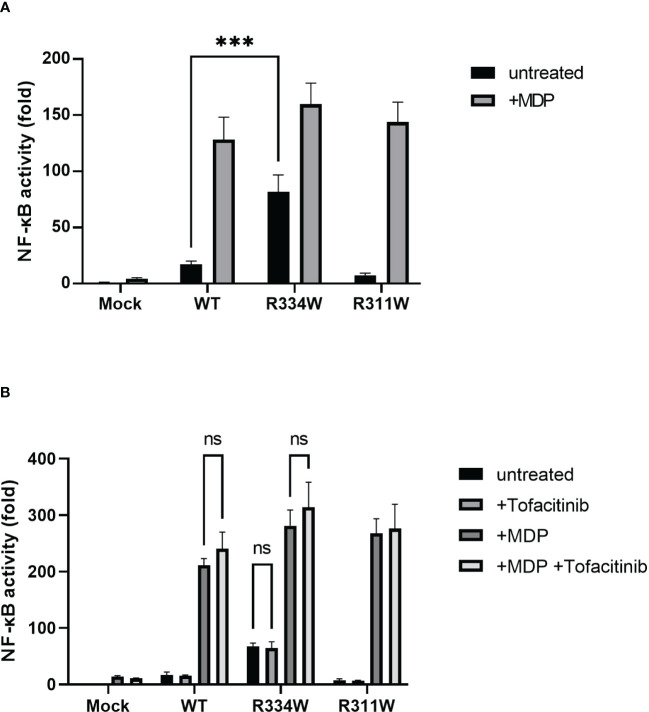
HEK293FT cells transfected with Blau syndrome-associated mutant *NOD2* showed ligand-independent NF-κB activation, as evaluated with an NF-κB luciferase reporter system. There was no downregulation by tofacitinib. The relative fold-change from the untreated condition of Mock (set as 1) is shown. Bars indicate means ± SDs (n=3). This test was repeated at least three times, and the results of one representative test are shown, but the same results were obtained each time. **(A)** Cells were treated with MDP (10 µg/mL) for 24 (h) **(B)** Cells were treated with MDP (10 µg/mL) with or without tofacitinib (1 uM) for 24 **(h)** ***p <.001, ns. not significant.

Zhang et al. (20) studied the efficacy of tofacitinib in three patients with Blau syndrome. They proposed a model in which JAK, which acts downstream of the cytokine receptor, was activated by a mutated NOD2. To explore this possibility, we first examined whether the JAK-STAT signaling pathway was involved downstream from NOD2. Towards that end, we used HEK293FT cells transiently transfected with a plasmid carrying *NOD2*. Here, p.R334W is a representative mutation in Blau syndrome and p.R311W is a nonpathological single nucleotide variant (SNV) (10). First, we performed dual-luciferase assays of NF-κB. The result was the same as our previous report (1, 10, 11). That is, in mutant-p.R334W transfected cells, NF-κB was spontaneously activated in an MDP-independent manner (**Figure 1A**). In contrast, WT and non-pathogenic SNV, R311W, as well as pathogenic p.R334W, increased luciferase activity in response to MDP.

Under the addition of tofacitinib, however, there was no attenuation of NF-κB activation in any type of NOD2-transfected cell, with or without the addition of MDP (**Figure 1B**). Those results indicated that the JAK-STAT pathway was not directly associated with the downstream pathway of NOD2, regardless of the presence or absence of NOD2 mutations associated with Blau syndrome.

**Figure 2:**
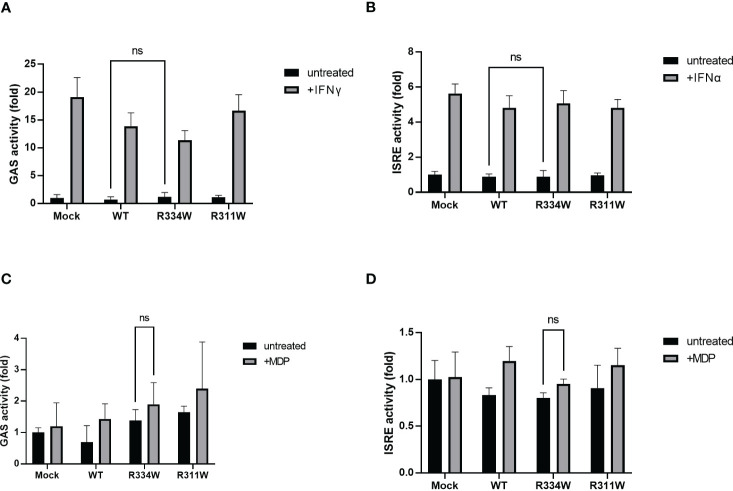
There was no significant difference in GAS or ISRE activation, as evaluated by a dual luciferase reporter system. The relative fold-change from the untreated condition of Mock (set as 1) is shown. Bars indicate means ± SDs (n=3). This test was repeated at least three times, and the results of one representative test are shown, but the same results were obtained each time. **(A)** Cells were treated with IFNγ (50 ng/mL) as a positive control for the activation of the GAS reporter. **(B)** Cells were treated with IFNα (1000 U/mL), as a positive control for the activation of the ISRE reporter. **(C, D)** Cells were treated with MDP (10 µg/mL) for 24 **(h)** Cells were treated with MDP (10 µg/mL) for 24 **(h)**, ns. not significant.

To ensure that the JAK-STAT pathway was not involved downstream from NOD2, we performed dual-luciferase assays of ISRE and GAS. When IFNα or IFNγ was added, activation of either ISRE or GAS was detected, respectively (**Figures 2A, B**). Those data confirmed that the assay system itself functioned properly. In both assays, we found no specific activation of ISRE or GAS in mutant-NOD2 transfected cells (**Figures 2C, D**). Even the stimulation of NOD2 with ligand MDP did not initiate ISRE or GAS activation. Taken together, we concluded that the JAK-STAT pathway is not directly involved downstream from NOD2.

### Tofacitinib suppresses NOD2 induction by IFNγ in iPS-ML

**Figure 3:**
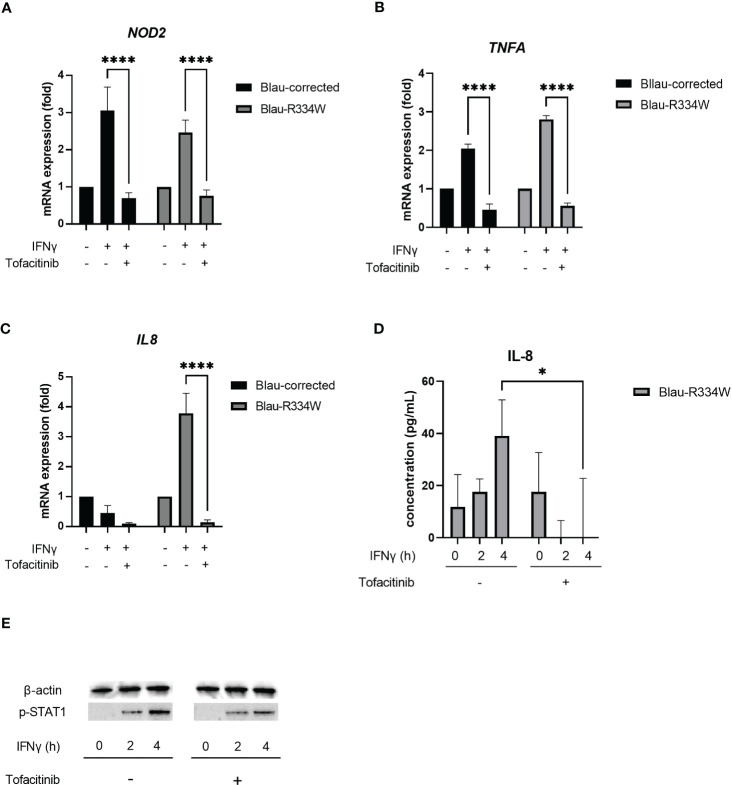
Tofacitinib downregulated NOD2 induction, inflammatory cytokine production and phosphorylation of STAT1 by IFNγ. This test was repeated at least three times, and the results of one representative test are shown, but the same results were obtained each time. Bars indicate means ± SDs (n=3). *p <.05, ****p <.0001. Quantitative RT-PCR validation of *NOD2* **(A)**, *TNFA* **(B)** and *IL8* expression **(C)** in the patient-derived iPS-MLs with corrected wild-type NOD2 (Blau-corrected) or disease-associated mutant *NOD2* (Blau-R334W). Cells were treated with IFNγ (50 ng/mL) for 6 h with or without tofacitinib (1 µM). In Quantitative RT-PCR, the relative fold-change from each untreated condition (set as 1) is shown. **(D)** Secretion of cytokine IL-8 from Blau-R334W evaluated by ELISA. **(E)** Phosphorylation of STAT1 (pSTAT1) by Western Blotting.

To evaluate the anti-inflammatory effect of tofacitinib in Blau syndrome, we focused our attention on the upstream pathway of NOD2. For this evaluation, we used iPS-ML cell lines established from a Blau syndrome patient; one line had the p.R334W mutation in *NOD2* and the other mutation was corrected back to WT by CRISPR-Cas9–mediated targeting. qPCR experiments in both iPS-ML cell lines showed that stimulation with IFNγ for 2 h induced *NOD2*, regardless of the presence or absence of the Blau syndrome-associated mutation (**Figure 3A**). Similar results were obtained in our previous experiments with macrophages generated by culturing iPS-ML cells in RPMI-1640 medium supplemented with 10% FCS containing 100 ng/mL M-CSF for 7 days (16, 23). The same results were observed when IFNγ was used to induce *NOD2* in iPS-ML cells established from the control iPS line 201B7E1 (data not shown). In the iPS-ML cells from 201B7E1, we previously confirmed that *NOD2* expression was induced by IFNγ using macrophages induced by further differentiation, similar to observations obtained with iPS-ML cells generated from Blau syndrome patient-derived iPS cells (16). These results suggested that it was possible to evaluate the effect of tofacitinib on the NOD2-mediated inflammatory pathway associated with Blau syndrome without differentiating iPS-ML cells further into macrophages.

In summary, tofacitinib suppressed the expression of *NOD2* induced by IFNγ in all types of cells, regardless of the presence or absence of the p.R334W mutation in *NOD2* (**Figure 3A**).

### Tofacitinib suppresses inflammatory cytokine production from iPS-ML with NOD2 mutation

Next, we examined the proinflammatory cytokines produced by iPS-ML cells. Along with the induction of *NOD2* expression by IFNγ treatment, *TNFA* expression was induced not only in cells with the p.R334W mutation associated with Blau syndrome, but also in WT cells (**Figure 3B**). This IFNγ-induced expression of *TNFA* was suppressed by tofacitinib.

In contrast, expression of *IL8*, also known as *CXCL8*, was observed only in mutant iPS-ML cells with the p.R334W mutation, suggesting that *IL8* is a suitable indicator of mutant NOD2 activation (**Figure 3C**). The increased expression of *IL8* was attenuated by the addition of 1 μM tofacitinib.

Upon induction of *IL8* expression, sufficient amounts of IL-8 were released into the cell supernatant in mutant iPS-ML cells 4 h after IFNγ treatment, and this production was suppressed by tofacitinib treatment (**Figure 3D**). When STAT1 phosphorylation (pSTAT1) was confirmed by western blotting, pSTAT1 was not observed before IFNγ stimulation in the mutant iPS-ML cells, but only after IFNγ treatment. This induction was observed to be suppressed by tofacitinib treatment, although not completely (**Figure 3E**). These results confirm our findings that tofacitinib reduces inflammatory cytokines in Blau syndrome by suppressing *NOD2* expression.

## Discussion

JAK inhibitors are commonly used for the treatment of inflammatory diseases such as rheumatoid arthritis. Recently, those inhibitors have attracted interest as therapies for inflammatory skin disorders, such as atopic dermatitis and psoriasis. Furthermore, there are reports suggesting their efficacy against granulomatous diseases (18, 19, 24–27) and, very recently, Blau syndrome (20).

The molecular mechanism of granulomatosis is unknown. One report showed that a patient with cutaneous sarcoidosis was successfully treated with tofacitinib. Moreover, staining for pSTAT1 and pSTAT3 was significantly higher in samples obtained from patients with sarcoidosis than in normal control skin samples. In addition, staining for pSTAT1 was strongest in the center of the granuloma and more prominent in macrophages than in T cells, whereas staining for pSTAT3 was strongest in the nuclei of lymphocytes within and between granulomas (14). However, precisely how a JAK inhibitor suppresses cutaneous granuloma formation is unclear.

**Figure 4:**
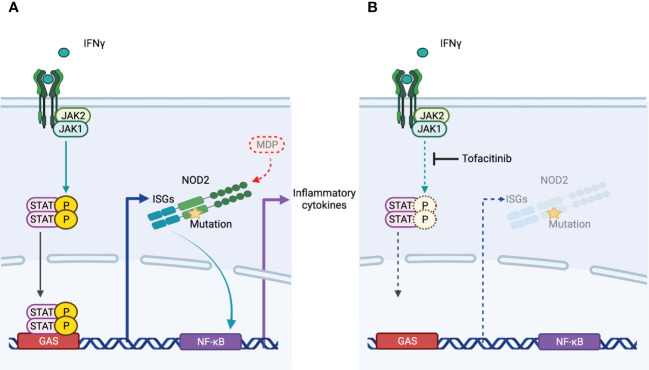
The mechanism of action of tofacitinib in Blau syndrome. **(A)** NOD2 expression is induced by IFNγ. NOD2 with mutations associated with Blau syndrome is activated in the absence of its ligand MDP, leading to increased transcription of NF-κB and production of proinflammatory cytokines. **(B)** Tofacitinib did not inhibit downstream signaling of NOD2. As an inhibitor of JAK, which acts downstream of IFNγ, resulting in the suppression of IFNγ-induced NOD2 expression, the inflammation of Blau syndrome is regulated.

As a NOD2-dependent autoinflammatory granulomatosis, Blau syndrome is a good model to investigate the mechanism of granuloma formation. We evaluated the effect of tofacitinib on Blau syndrome associated with pro-inflammatory responses. Tofacitinib did not suppress the increased transcriptional activity of NF-κB induced by mutant NOD2. In contrast, IFNγ induced *NOD2* expression both in the presence and absence of mutations. Instead, expression of *NOD2* results in the production of pro-inflammatory cytokines by an autoinflammatory mechanism in cells with mutant *NOD2*. Tofacitinib showed anti-inflammatory effects by suppressing the expression of *NOD2*. This suggests that tofacitinib will not affect granulomas once they have formed. However, it is unclear how granulomas are maintained. If persistent inflammation is required, this drug may be effective in some situations by suppressing *NOD2* expression and preventing the development of inflammation. In Blau syndrome, the skin rash goes through periodic exacerbations and remissions over a period of several months, with some cases spontaneously regressing with age. Thus, even if the therapeutic strategy is to suppress NOD2 expression, if it is effective in preventing inflammatory flare-ups, it may be effective in preventing the maintenance of granulomas as a result. Based on our experimental results and these discussions, we present the mechanism of action of tofacitinib in Blau syndrome as **Figure 4**.

Precisely how NOD2 activation induces granuloma formation in Blau syndrome remains to be elucidated. In this study, we focused on *ex vivo* studies using a luciferase assay system overexpressing the NOD2 mutant and iPS-ML cells differentiated from iPS cells derived from a Blau syndrome patient. In this respect, it would be of interest to isolate and culture monocytes from the blood of patients and perform similar experiments. However, most patients with Blau syndrome are children, and frequent invasive testing is not practical. The fact that we were able to elucidate the effects of tofacitinib without performing invasive procedures is an advantage of using patient-derived iPS cells.

The second limitation of this study is that a variety of immune cells, including cells resident in the tissue, may be involved in the formation of granulomas. Therefore, it will be necessary to examine whether tofacitinib affects immune cells within the granuloma, especially those that may produce IFNγ in the tissue. In addition, as an indicator of inflammatory cytokines in this study we used IL-8, a chemokine that induces neutrophil migration. However, the extent to which IL-8 and neutrophil migration are actually involved in the formation of granulomas that characterize Blau syndrome has not yet been addressed.

In this study, we demonstrated that tofacitinib suppresses inflammatory cytokine production by inhibiting the expression of *NOD2*. We hope that tofacitinib will become a treatment option for Blau syndrome in the future and that the mechanism of granuloma formation will be elucidated.

## Data availability statement

The original contributions presented in the study are included in the article/supplementary materials. Further inquiries can be directed to the corresponding author.

## Ethics statement

The studies involving human participants were reviewed and approved by CiRA18-06 in Kyoto University and 2017104 in Kansai Medical University. Written informed consent to participate in this study was provided by the participants’ legal guardian/next of kin.

## Author contributions

YU, RT-I, and NK designed the experiments. MKS and HT supported the planning and validated the strategy. YU and RT-I performed experiments. All authors were involved in drafting the article or revising it critically for important intellectual content, and all authors approved the final version to be published.
